# The Expression Patterns of Antibody–Drug Conjugate‐Related Markers in Urothelial Carcinoma With Histological Variants

**DOI:** 10.1002/mco2.70865

**Published:** 2026-07-15

**Authors:** Xingliang Tan, Xinpei Deng, KunYang Wang, Zhiming Wu, Yanjun Wang, Chichen Zhang, Qianghua Zhou, Yun Cao, Neng Jiang, Kai Yao

**Affiliations:** ^1^ Sun Yat‐sen University Cancer Center Guangzhou China; ^2^ Guangdong Provincial Clinical Research Center for Cancer State Key Laboratory of Oncology in Southern China Guangzhou China

1

Dear Editor:

Urothelial carcinoma (UC) is a common genitourinary malignancy and places a considerable burden on global public health, with more than 220,000 deaths worldwide in 2025 [1]. Although the majority of histological subtype is conventional UC, approximately 30% of tumors have histological variants, especially in advanced disease. Notably, UC with divergent differentiations has the potential to be aggressive and resistant to chemotherapy and immunotherapy, which is associated with poor survival of patients and poses substantial challenges for clinical treatment. Recently, UC care is moving toward precision oncology owing to the remarkable efficiency, low toxicity, and booming development of antibody‐drug conjugates (ADCs). For instance, compared with chemotherapy, the Nectin4‐targeted ADC enfortumab vedotin (EV) with pembrolizumab extends the median OS from 16.1 months to 31.5 months in la/mUC [2]. Disitamab vedotin (DV) plus toripalimab increased overall survival from 16.9 to 31.5 months [3]. BL‐B01D1, a first‐in‐class EGFR/HER3 bispecific ADC, had an objective response rate of 43.5% and a disease control rate of 91.3% [4]. Trop2‐targeted sacituzumab govitecan and HER3‐tageted AMT‐562 have broadened the spectrum of treatment options. However, the expression patterns of target genes in different histological variants remain unclear, and studies incorporating a systematic, large‐sample‐size, Asian population‐based database are lacking. A more comprehensive understanding of the heterogeneous expression of genes involved in divergent differentiation might provide guidance for therapeutic selection and optimization.

In this study, 354 UC patients with divergent differentiation (>10% areas) were enrolled. The clinical characteristics information was described in the Supporting Information. Among the patients, 49 patients had multiple types of differentiation, and 405 cases of histological variants were analyzed. Overall, squamous differentiation was the most common subtype of divergent differentiation accounting for up to 48.4% (196/405), followed by 24.7% glandular, 9.1% sarcomatoid, 6.2% micropapillary, 6.2% plasmacytoid, and 5.4% neuroendocrine variants (Table 1). The expressions of HER2, Nectin4, Trop2, HER3, and EGFR were detected by immunohistochemistry (IHC), and the procedure, scoring criteria, and antibody details were summarized in the Supporting Information.

**TABLE 1:** The expression patterns of ADC‐related markers in urothelial carcinoma with histological variants.

Histological variants (*n* = 405)	HER2 Expression				Nectin4 Positive	Trop2 Positive	EGFR Positive	HER3 Positive	Triple‐ Negative	Penta‐Negative
	0+	1+	2+	3+						
Squamous differentiation (*n* = 196)	145/196 (73.98%)	24/196 (12.24%)	21/196 (10.71%)	6/196 (3.06%)	172/196 (87.76%)	169/196 (86.22%)	189/196 (96.43%)	70/196 (35.71%)	10/196 (5.1%)	0/196 (0.0%)
Glandular differentiation (*n* = 100)	38/100 (38.00%)	32/100 (32.00%)	14/100 (14.00%)	16/100 (16.00%)	47/100 (47.00%)	75/100 (75.00%)	46/100 (46.00%)	52/100 (52.00%)	2/100 (2.0%)	0/100 (0.0%)
Micropapillary differentiation (*n* = 25)	3/25 (12.00%)	7/25 (28.00%)	11/25 (44.00%)	4/25 (16.00%)	19/25 (76.00%)	22/25 (88.00%)	13/25 (52.00%)	13/25 (52.00%)	0/25 (0.0%)	0/25 (0.0%)
Sarcomatoid differentiation (*n* = 37)	32/37 (86.49 %)	3/37 (8.11%)	2/37 (5.41%)	0/37 (0.00%)	8/37 (21.62%)	29/37 (78.38%)	16/37 (43.24%)	7/37 (18.92%)	6/37 (16.2%)	5/37 (13.5%)
Small‐cell neuroendocrine differentiation (*n* = 22)	18/22 (81.82%)	4/22 (18.18%)	0/22 (0.00%)	0/22 (0.00%)	8/22 (36.36%)	8/22 (36.36%)	5/22 (22.73%)	4/22 (18.18%)	8/22 (36.4%)	5/22 (22.7%)
Plasmacytoid differentiation (*n* = 25)	4/25 (16.00%)	4/25 (16.00%)	11/25 (44.00%)	6/25 (24.00%)	18/25 (72.00%)	17/25 (68.00%)	10/25 (40.00%)	17/25 (68.00%)	0/25 (0.0%)	0/25 (0.0%)

*Note*: ADC‐related target gene expression was evaluated by immunohistochemistry. 405 histological components from 354 patients, including 291 bladder cancer and 63 upper tract urothelial carcinoma. HER2 high expression refers to IHC 2/3+. Triple‐negative refers to tumors negative for HER2, Nectin4, and Trop2. Penta‐negative refers to tumors negative for HER2, Nectin4, TROP2, EGFR, and HER3. ADC: antibody‐drug conjugate.

With respect to squamous differentiation, 96.4% of the patients were EGFR positive, 87.8% were Nectin4 positive, and 86.2% were Trop2 positive. However, HER2 expression was relatively low, with a 26.0% positive rate (IHC: 1–3+), and only a 13.8% high expression rate (IHC: 2/3+) was detected. In total, 35.7% of UC patients had HER3‐positive expression that was not significantly correlated with EGFR or HER2 overexpression. Therefore, Nectin4 and Trop2 served as actionable therapeutic targets, and EV might be the optimal choice while SG and DV can be used as sequential therapies or for EV‐resistant UC with squamous differentiation.

Glandular differentiation is the second most common histological variant in UC. In all 100 samples, the positive expression rates of HER2, Nectin4, Trop2, EGFR, and HER3 were 62.0%, 47.0%, 65.0%, 46.0%, and 52.0%, respectively. High HER2 expression was observed in 30% of patients with UC with glandular differentiation. Only 2% (2/100) of patients simultaneously lacked HER2, Nectin4, and Trop2 expression. Therefore, comprehensive testing of ADC‐related targets is recommended, and appropriate strategies for clinical practice should be selected.

Micropapillary differentiation strongly increased HER2 and Trop2 expression. Among the 25 samples analyzed, 88.0% had positive Trop2 expression, and 88.0% had positive HER2 expression, among which 60.0% had high HER2 expression (IHC: 2–3+). In addition, 76.0% of the micropapillary differentiated tumors were positive for Nectin4 expression, and 52.0% patients had EGFR and HER3 positive expressions, respectively. Micropapillary differentiation often resulted in the expression of multiple targets, and none of the samples were triple‐negative.

Sarcomatoid differentiation revealed that only Trop2 was a potential ADC‐related therapeutic target, with a positive expression rate of 78.4% (29/37). However, HER2 and Nectin4 expressions were less common in sarcomatoid variants, with positive rates of only 13.5% and 21.6%, respectively. EGFR expression was detected in 43.2% of tumor samples and could be a direction for future clinical trials. HER3 expression was also relatively low, with a positive rate of only 18.9%. Up to 18.9% of patients simultaneously lack Nectin4, HER2, and Trop2 expressions. Therefore, Trop2‐ADCs might represent an effective targeted therapeutic approach, especially for patients with HER2/Nectin4 double‐negative sarcomatoid differentiation.

Pure small‐cell neuroendocrine tumors or tumors mixed with high‐grade UC with sarcomatoid divergent differentiation is rare malignancies with the potential to aggressively metastasize early and poor prognosis. The expression of ADC‐related markers was low, with a positive rate of only 18.2% for HER2, 36.4% for Nectin4, 36.4% for Trop2, 22.7% for EGFR, and 18.2% for HER3. None of the small‐cell neuroendocrine variants had high HER2 expression. The triple‐negative rate was 36.4% (8/22), and even 22.7% (5/22) of the patients did not show the expression of all five targets. Therefore, PE chemotherapy (cisplatin and etoposide) is still currently the best regimen, and the available targeted therapies need further exploration.

Plasmacytoid differentiation is characterized by high expression of HER2. Among the 25 samples, 84.0% (21/25) UC patients with plasmacytoid differentiation had high HER2 expression, and up to 68.0% (17/25) had high HER2 expression. The Nectin4‐, Trop2‐, EGFR‐ and HER3‐positive rates were 72.0%, 68.0%, 40.0%, and 68.0%, respectively. All patients expressed at least one target, and HER2‐targeted ADCs such as DV could be an available treatment.

Recently, ADCs have made breakthrough advances with favorable safety profiles, but the efficacy varies substantially across different histological subtypes. In squamous differentiation, Nectin4 and Trop2 are highly expressed, while HER2 expression is low, suggesting that EV may be the most suitable therapeutic option. The UNITE multicenter study, which retrospectively involved 633 advanced UC patients, demonstrated that EV had 55% (31/56) ORR rate and 12.7 months of median overall survival, with comparable outcomes to those of pure UC [2]. In contrast, micropapillary differentiation and plasmacytoid differentiation were HER2‐enriched phenotypes, and approximately 25%–56% of patients had HER2 overexpression [5]. Similarly, our study revealed that the HER2‐positive rates were 88.0% and 84.0%, with 60% and 68.0% overexpression rates in patients with UC with micropapillary differentiation and plasmacytoid differentiation, respectively, indicating a potential benefit from HER2‐targeted ADC therapy. Glandular differentiation often has multiple targets, and an effective combination of ADC therapy and sequential regimens remains to be determined through clinical research. Although ADC‐based therapies are likely to remain effective for the majority of urothelial carcinomas, the loss of multiple ADC targets is particularly enriched in sarcomatoid and small‐cell differentiation. We found that UC with sarcomatoid and small‐cell differentiation without HER2, Nectin4, and Trop2 expression (triple‐negative) was detected in 18.9% of patients with sarcomatoid variants and 36.4% of patients with neuroendocrine variants and even in 13.5% and 22% of patients without EGFR and HER3 expression, respectively. Therefore, additional comprehensive sequencing is recommended to provide more information for clinical decision‐making in patients with sarcomatoid and small‐cell neuroendocrine differentiation. In addition, traditional chemotherapy remains the foundation of treatment.

Although our current analysis is limited to descriptive expression profiling without further validation of therapeutic efficacy, the results still support a target‐based therapeutic strategy to treat with diversity histological variants in UC. Besides, the expression patterns of several rare variants, such as nested, tubular, lymphoepithelioma‐like, and clear cell differentiation, require multicenter collaboration for exploration.

## Author Contributions

Kai Yao and Neng Jiang had full access to all the data in the study and they take the responsibility for the integrity of the data and the accuracy of the data analysis. Study concept and design: Xingliang Tan, Xinpei Deng, and Kun Yang Wang. Acquisition of data: Zhiming Wu. Analysis and interpretation of data: Chichen Zhang and Qianghua Zhou. Statistical analysis: Yanjun Wang and Yun Cao. Drafting of the manuscript: Xingliang Tan. Critical revision of the manuscript: Neng Jiang. Funding acquisition: Xingliang Tan and Kai Yao. All authors have read and approved the final manuscript.

## Funding

This study was supported by the National Natural Science Foundation of China General Program (No. 82472805), GuangDong Basic and Applied Basic Research Foundation grant (2024A1515013007), and the East Clinical Center of Oncology Foundation grant (No. ECCO‐KY‐25007) awarded to Kai Yao, and Guangdong Medical Science and Technology Research Fund (No. A2024379) awarded to Yanjun Wang. This study was also supported by the Young Scientists Fund of the National Natural Science Foundation of China (No.82503944) awarded to Xingliang Tan.

## Ethics Statement

Research approval was obtained from Sun Yat‐sen University Cancer Center Ethics Committees (B2024‐058‐01) in accordance with the Declaration of Helsinki. Informed consent was waived due to the retrospective nature of the investigation.

## Conflicts of Interest

The authors declare no conflicts of interest.

## Supporting information




**Supporting Information**: mco270865‐sup‐0001‐SuppMat.docx

## Data Availability

The datasets supporting the conclusions of this article are available from the corresponding author upon reasonable request. The authenticity of this article has been validated by uploading the key raw data onto the Research Data Deposit platform (www.researchdata.org.cn) with the Number of RDDB2026656414.
