# Supplementary material for: The Expression Patterns of Antibody–Drug Conjugate‐Related Markers in Urothelial Carcinoma With Histological Variants
Source: MedComm (2020). 2026 Jul 15;7(8):e70865. doi: 10.1002/mco2.70865 (PMC13370099; doi:10.1002/mco2.70865)
Supplement: Supplementary file 1 — Supporting Information: mco270865‐sup‐0001‐SuppMat.docx [file MCO2-7-e70865-s001.docx]

**Supplementary Materials for**

The expression patterns of antibody-drug conjugates related markers in urothelial carcinoma with histological variants

Xingliang Tan^1,2*^, Xinpei Deng^1,2*^, KunYang Wang^1,2*^, Zhiming Wu^1,2^, Yanjun Wang^1,2^, Chichen Zhang^1,2^, Qianghua Zhou^1,2^, Yun Cao^1,2#^, Neng Jiang^1,2#^, Kai Yao^1,2#^

^1^Sun Yat-sen University Cancer Center, Guangzhou 510060, China

^2^Guangdong Provincial Clinical Research Center for Cancer, State Key Laboratory of Oncology in Southern China, Guangzhou 510060, China

^#^Corresponding authors:

Yun Cao, E-mail: [caoyun@sysucc.org.cn;](mailto:wangyj@sysucc.org.cn;)

Neng Jiang, E-mail: [jiangneng@sysucc.org.cn;](mailto:wangyj@sysucc.org.cn;)

Kai Yao, E-mail: yaokai@sysucc.org.cn

**The file includes:** Materials and methods

**Materials and methods**

Patients cohort

This retrospective study enrolled 354 advanced urothelial carcinoma (UC) patients including 291 cases of bladder cancer and 63 cases of upper tract urothelial carcinoma (UTUC) at Sun Yat-sen University Cancer Center (SYSUCC). Patient eligibility criteria included (1) detailed clinical data and available tumor sections for immunohistochemistry (IHC) staining (2) pathologically confirmed urothelial carcinoma with histological variants (>10% area) or pure squamous cell carcinoma, adenocarcinoma, sarcoma and neuroendocrine carcinoma. Pathology information was individually determined by experienced pathologists at SYSUCC on the basis of the 8th edition of the AJCC TNM Staging System. The exclusion criteria were insufficient tumor tissue, poor tissue fixation, and the absence of clinicopathologic features.

Patient Characteristics

A total of 354 patients referred to Sun Yat-Sen University Cancer Center between from January 1, 2013 to July 31, 2024 were included in the study. Among them, 291 patients were bladder cancer and 63 were upper tract urothelial carcinoma. In total, 109/291(37.5%) bladder cancer and 15/63 (23.8%) UTUC patients received transurethral resection of bladder tumour or extended tumor resection and the other 62.5% and 76.2% patients performed with radical cystectomy or nephroureterectomy. The pathological stages of pTa/T1, pT2, pT3 and pT4 were 25.4%, 19.8%, 35.3% and 19.5% respectively. 71.8% patients had no regional lymph node metastasis.

Assessment of HER2, HER3, Nectin4, Trop2 and EGFR expression

In brief, 4-µm paraffin-embedded, well-preserved tumor sections were retrieved for regular pathological examination by hematoxylin and eosin (HE) staining. The expression of HER2, HER3, Nectin4, Trop2 and EGFR on the histological differentiation areas of the tumor section was evaluated by IHC staining. The area of histological variants should account for more than 10% of the entire tumor section. If multiple differentiatial components are present, each should be scored separately. HER2 expression was automatically detected by an anti-HER2/neu (4B5) rabbit monoclonal antibody (Ventana; Roche Diagnostics) on the Ventana Bench Mark XT platform. All procedures were set and reproducible on the basis of the Ventana platform and represented the gold standard for anti-HER2 therapies. The scoring criteria were based on the consensus of the Expert Committee on Urothelial Carcinoma of the Chinese Society of Clinical Oncology in 2021. HER2 2+ or 3+ was considered to indicate high HER2 expression. HER3, Nectin4 and Trop2 were evaluated by H-scores (range 0-300, rank 0 to 3+) multiplied by the extent and intensity of the staining. H-scores >15 were considered to indicate positive expression (>15 and ≤100 was 1+, >100 and ≤200 was 2+, and >200 and ≤300 was 3+). The antibodies used were as follows: anti-HER3 (Cell Signaling Technology, HER3/ErbB3, #12708), anti-Nectin4 (Abcam, ab192033), anti-Trop2 (Signaling Technology, TACSTD2/TROP2, #76730) and anti-EGFR (Signaling Technology, #4267). The scoring was performed by two pathologists (JN and CKM) who were blinded to the clinical data. If the results were inconsistent, the slides were reexamined, and a consensus was reached after discussion.

Statistical analyses

Only descriptive statistical analysis was adopted in this study. Continuous variables were summarized as mean±standard deviation or median (interquartile range), and categorical variables were reported as counts and corresponding percentages (n, %).
